# StBIN2 Positively Regulates Potato Formation through Hormone and Sugar Signaling

**DOI:** 10.3390/ijms242216087

**Published:** 2023-11-08

**Authors:** Jie Liu, Chengcheng Cai, Shifeng Liu, Liqin Li, Qiang Wang, Xiyao Wang

**Affiliations:** 1 College of Agronomy, Sichuan Agriculture University, Chengdu 611130, China; liujie-116@foxmail.com (J.L.); cccqyj@hotmail.com (C.C.); 2020101001@stu.sicau.edu.cn (S.L.); liliqin88@163.com (L.L.); 2 Potato Research and Development Center, Sichuan Agricultural University, Chengdu 611130, China

**Keywords:** potato, tuber formation, StBIN2, hormones, sugar, brassinosteroid

## Abstract

Potato is an important food crop worldwide. Brassinosteroids (BRs) are widely involved in plant growth and development, and BIN2 (brassinosteroid insensitive 2) is the negative regulator of their signal transduction. However, the function of BIN2 in the formation of potato tubers remains unclear. In this study, transgenic methods were used to regulate the expression level of *StBIN2* in plants, and tuber related phenotypes were analyzed. The overexpression of *StBIN2* significantly increased the number of potatoes formed per plant and the weight of potatoes in transgenic plants. In order to further explore the effect of StBIN2 on the formation of potato tubers, this study analyzed BRs, ABA hormone signal transduction, sucrose starch synthase activity, the expression levels of related genes, and interacting proteins. The results show that the overexpression of *StBIN2* enhanced the downstream transmission of ABA signals. At the same time, the enzyme activity of the sugar transporter and the expression of synthetic genes were increased in potato plants overexpressing *StBIN2*, which also demonstrated the upregulation of sucrose and the expression of the starch synthesis gene. Apparently, StBIN2 affected the conversion and utilization of key substances such as glucose, sucrose, and starch in the process of potato formation so as to provide a material basis and energy preparation for forming potatoes. In addition, StBIN2 also promoted the expression of the tuber formation factors StSP6A and StS6K. Altogether, this investigation enriches the study on the mechanism through which StBIN2 regulates potato tuber formation and provides a theoretical basis for achieving a high and stable yield of potato.

## 1. Introduction

Potato (*Solanum tuberosum* L.) has strong adaptability, a high yield, and high nutritional value and is the main food source and economic source in high-altitude mountain areas [1,2]. The most direct factor influencing potato yield is tuber formation. At present, scholars mainly study the generation of potato stolons, the transformation of stolons into tubers, and the expansion of stolons in sensing and adapting to short day conditions [3,4]. These are also the three parts of the tuber formation process, which is a pivotal factor affecting potato yield. Tuber formation occurs in cold environments with low nitrogen contents and short periods of sunshine, and these processes are regulated by many internal and external sources [5,6]. Therefore, in potato production, it is crucial to analyze the mechanism of tuber formation and its regulation path.

Understanding the tuber formation process of potato has been the goal of many plant biologists around the world. The graft transmission characteristics of the potato tuber’s signal and the accompanying morphological changes in potato plants have shown in many studies that plant hormones may be the stimulating factors in the tuber induction process [7], and the exogenous application of ABA, BRs, and other hormones plays an important role in regulating potato yield. One of the other important factors in determining yield is the accumulation of starch [8,9,10]. During the development of tubers, sucrose 2, as an indispensable metabolic signal, regulates the content of starch [11], and the number of tubers can be increased by increasing the accumulation of sugar or by applying exogenous sucrose [12]. In addition, sugars and plant hormones regulate each other’s metabolic, transport, and signaling pathways, and their interaction plays a key role in tuber formation [13]. In addition, tuber formation is strongly influenced by transcription factors [14], and its further development is the result of the synergistic effect of multiple internal and external factors [15], including the photoperiod, temperature, light intensity, and nitrogen supply [16].

Brassinosteroids (BRs), plant sterol hormones, play an important role in plant growth and development [17]. BRs can regulate yield in tomato, rice, and potato via the concentration effect. The exogenous application of BRs and upregulation of BR synthesis gene expression can affect yield [18,19,20]. In recent years, the research on BRs has focused on their synthesis and signal transduction process. BIN2, a protein kinase, as the key negative regulator of BR signal transduction [21], plays an important role in the growth and development of various crops. However, the influence of BIN2 on yield has not been fully studied. Research studies on BIN2 mainly focus on the effect of BIN2 on hormone signaling and meristem response to stress, thus leading to the hypothesis that a high yield can be obtained under stress [22,23]. Therefore, it is of great significance to explore the role of BIN2 in the yield formation of potato.

Studies on the interaction between BIN2 and plant hormones have been ongoing for a long time. BIN2 plays an important role in linking BRs and ABA hormone signaling pathways; BIN2, ABI5 and SnRK2s work together to participate in various physiological processes [24]. In *Arabidopsis thaliana*, BIN2 interacts with various hormones and sugar signals to participate in stress response and plant growth [25]. The downstream transcription factor BZR1 of BIN2 can interact with β-amylase to form a complex and at the same time regulate the BR signaling pathway and the utilization of starch degradation [26,27], and the upstream inhibitory proteins BSK8 and BSL2 regulate the phosphorylation and enzyme activity of SPS [28]. In addition, sucrose phosphosynthase is a substrate for the SnRKs family of potential BIN2 interaction factors [29,30]. Several members of the S6K and FT (FLOWERING LOCUS T) family, which are very important for tuber development, are potential interaction factors of BIN2 [31,32,33]. ABI5 and SP6A (a FT family member) interact with each other to regulate potato tuber development [34]. In addition, the sugar transporter SWEET11 potentially interacts with SP6A [35].

In this study, to clarify the role of StBIN2 and its related regulatory mechanism in tuber formation via the genetic transformation of potato, transgenic potato plants with *BIN2* overexpression were generated, and their tuber formation phenotype was calculated. The key genes that may co-regulate tuber formation with BIN2 were initially screened. The activity and gene expression levels of related proteins were determined, and protein interaction experiments were carried out to elucidate the effect of StBIN2 on potato formation, providing a theoretical basis for increasing potato yield and molecular breeding.

## 2. Results

### 2.1. Bioinformatics Analysis of StBIN2 and the Expression Level of Transgenic Lines

**Figure 1:**
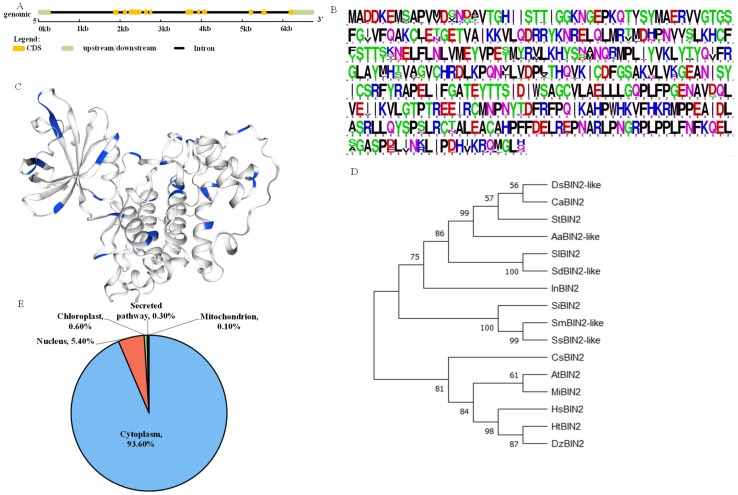
Prediction of subcellular location, conservative alignment of protein sequences, and evolutionary tree analysis. (**A**) Gene structure analysis. (**B**) Protein sequence conservative alignment, and different colors are used to distinguish the types of amino acids. (**C**) Prediction of the tertiary structure of StBIN2 protein, with Ser/Thr structure represented in blue. (**D**) Evolutionary tree analysis. (**E**) Prediction of subcellular localization.

A bioinformatic analysis showed that *StBIN2* is located on chromosome 3, containing 12 exons and 11 introns, and the coding sequence is 1149 bp, encoding a protein composed of 382 amino acids (Figure 1A). As shown in Figure 1B,C, BIN2 is a highly conserved Ser/Thr kinase, as per a protein sequence conservation alignment analysis. By constructing a phylogenetic tree of StBIN2 and its homologous proteins, as is shown in Figure 1C, it was found that potato StBIN2 is closely related to other *Solanaceae* plants, tomato and pepper. The subcellular localization prediction results showed that the StBIN2 protein was mainly located in the cytoplasm (93.50%) and nucleus (5.4%), accounting for 98.90% of the total localization (WoLF PSORT), as shown in Figure 1D.

An analysis of the *cis*-elements of *StBIN2* showed that the upstream promoter sequence of this gene contained multiple hormone- and photoresponsive elements and transcription factor binding sites, as shown in Table 1, including an ABA response element, ABRE (−50), the auxin response elements TGA-box A (−23) and TGA-element (−1296), the gibberellin response elements TATC-box (−643, −871) and P-box (−64, −82, −512), MeJA, salicylic acid, G-box, GA-motif, Box4, meristem related and other photoresponsive elements (other promoter *cis*-elements not related to this study are not listed in the table). It is predicted that this gene may be regulated by hormones and light signals.

**Figure 2:**
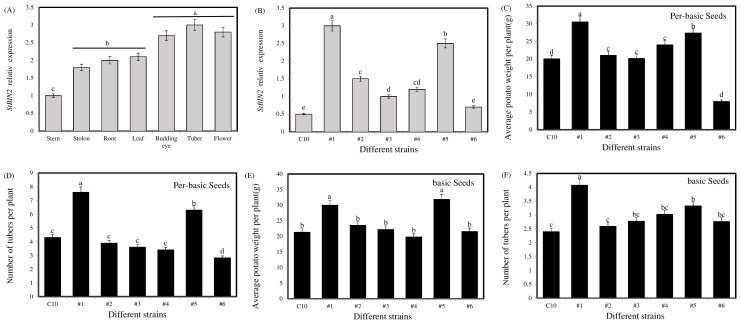
*StBIN2* expression levels and phenotypic analysis of StBIN2 transgenic lines. (**A**) *StBIN2* gene expression in different potato tissues. (**B**) Identification of *StBIN2* expression in different potato transgenic lines. (**C**) The average number of tubers per plant from pre-basic seed. (**D**) The average weight of potato per plant from pre-basic seed. (**E**) The average number of tubers per plant from basic seed. (**F**) Average potato weight per plant from basic seed. Different small letters represent significant differences (*p* < 0.05).

A qRT-PCR analysis confirmed that St*BIN2* was highly expressed in developing tubers (Figure 2A). The transgenic potato plants were generated with *StBIN2* overexpression, and six transgenic lines were selected to detect the *StBIN2* expression levels (Figure 2B) and count the number of tubers (Figure 2C–F). Overexpression line #1 showed a significant increase in the number of pre-basic seed tuber per plant (76%) and tuber weight (49%). The number of basic potato seeds per plant was increased by 69%, and the weight of the potato seeds per plant was increased by 40%. In conclusion, StBIN2 can indeed promote the number of tuber sets, and the promotion effect is positively correlated with the expression level to a certain extent. Hence, line #1 was selected as our follow-up experimental material because it had the highest level of expression of *StBIN2* and the most significant phenotype.

### 2.2. Effects of StBIN2 on the Formation of Potato Tubers

#### 2.2.1. Phenotypic Analysis of the Effects of StBIN2 on Tuber Formation in Potato

**Figure 3:**
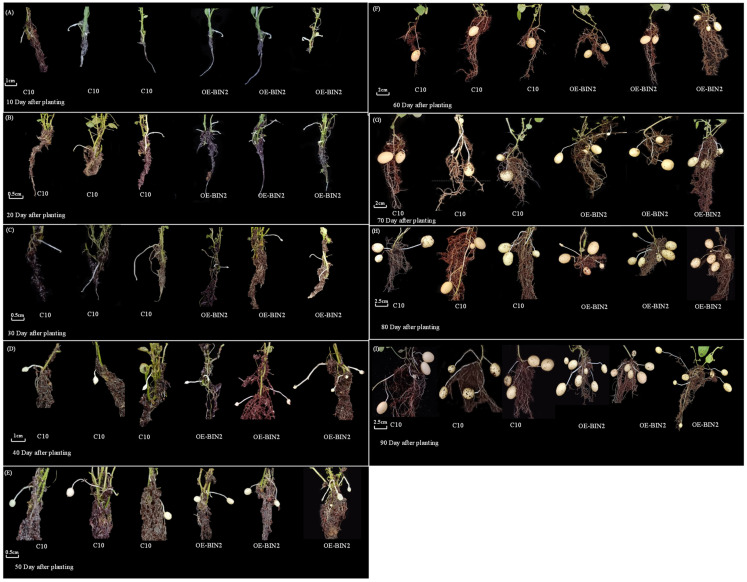
Effect of StBIN2 on the potato tuber phenotype. (**A**–**I**) Tuber formation phenotypes of tissue-cultured seedlings after 10–90 days of being planted.

**Figure 4:**
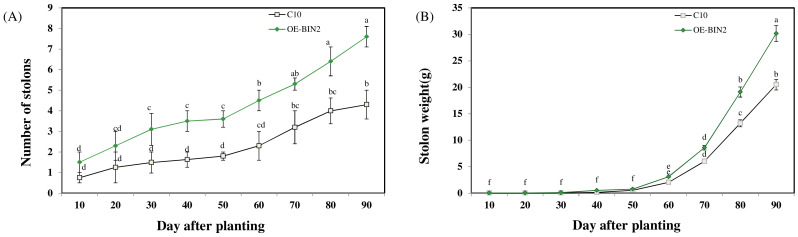
Effect of StBIN2 on the number and weight of potato stolons. (**A**) Number of stolons. (**B**) Stolon weight. Different small letters represent significant differences (*p* < 0.05).

To investigate the growth process of the per-basic seeds, we performed a sampling analysis of the intergrowth period for #1, and the phenotype is shown in Figure 3A–I. A significance analysis of several periods (Figure 4A,B) showed that the number of tubers formed per plant from an overexpressed line was significantly higher than the wild-type plants during the 30th–60th day and 80th–90th day after planting (samples were analyzed every 10 day). The analysis revealed the potato weight per plant, with significant differences between the two lines on the 80th day. From the 40th to 50th day, the number of stolons did not increase significantly, but the weight of the tubers increased significantly, which could be inferred as a period of concentrated expansion for the stolons.

#### 2.2.2. StBIN2 Regulates BRs and ABA Signal Transduction during Seed Potato Formation

**Figure 5:**
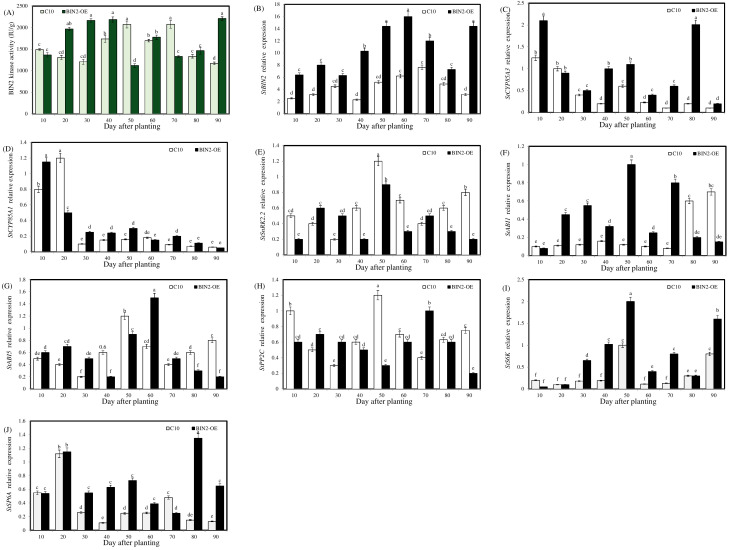
BIN2 kinase activity during potato tuber formation and the effect of StBIN2 on the expression of genes related to BRs and ABA signaling pathways during potato tuber formation. (**A**) BIN2 kinase activity. (**B**–**J**) The relative expression levels of *StBIN2, StCYP85A3*, *StCYP85A1*, *StSnRK2.2*, *StABI1*, *StABI5*, *StPP2C*, *StS6K*, and *StSP6A,* respectively. Different lowercase letters represent significant differences (*p* < 0.05).

As shown in Figure 5A, the activity of the BIN2 protein in the overexpressed lines increased significantly on the 30th day after planting, and the first kinase activity peak occurred earlier than that of the wild-type (C10) potatoes. Combined with the result that the overexpressing potatoes in Section 2.2.1 showed a significant increase in the number of potato stolons on the 30th day, BIN2 kinase activity played an important role in the process of stolonization.

Over the whole growth period, the expression level of *StBIN2* was kept higher in the transgenic line than the wild type (C10) (Figure 5B); especially in the process over the 40th–60th day (the concentrated period of stolon tip expansion), the expression level of *StBIN2* in blocks of overexpressing potato was significantly higher than that of the C10 potatoes. Therefore, our next data analysis work mainly focused on the 40th–60th day. It is worth noting that the expression level of the BR synthesis gene (*StCYP85A3*) in overexpressed plants was higher than in the C10 potatoes (Figure 5C), presumably because BR synthesis was increased in response to the inhibition of signal transduction. There was no significant difference in the expression of another BRs synthetic gene (*StCYP85A1*) in the tuber-concentrated potato-forming stage (Figure 5D).

The ABA signaling genes (Figure 5E–H) showed differential expression levels from 40 to 60 days, and the expression level of *StPP2C* showed a significant difference only on the 50th day. The gene expression level of *StSnRK2.2* was significantly lower than that of the C10 potatoes at the 40th–50th day but higher than at the 20th–30th day, indicating that the effect of StBIN2 on *StSnRK2.2* was not completely consistent in the process of potato formation. *StABI1* expression was significantly higher than that of the C10 potatoes. The expression level of *StABI5* was significantly higher than that of overexpression on the 60th day, and the peak value was the highest.

As shown in Figure 5J, during the critical period of stolon formation (the 30th–60th day), the expression level of *StSP6A*, a key gene affecting potato formation, was higher than that of the C10 potatoes. Combined with the effect of this gene, it was speculated that the overexpression of *StBIN2* promoted the number of tubers formed by regulating the expansion of the top of the stolon instead of the stolon growing upward into a stem. The expression level of *S6K* in the *StBIN2* overexpressing potatoes was significantly higher than that of the C10 potatoes during the period of concentrated stolon occurrence (30–60 d) (Figure 5I).

#### 2.2.3. StBIN2 Regulates the Effect of Sugar on Seed Potato Formation

**Figure 6:**
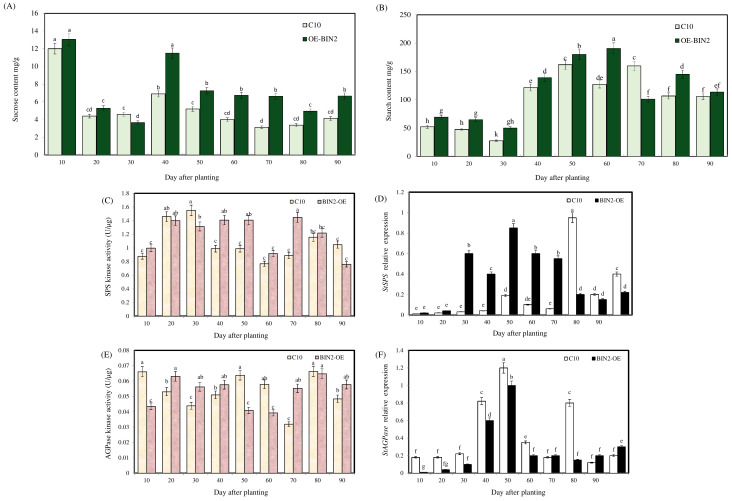
Effect of StBIN2 on the starch and sucrose synthesis of potato during growth period. (**A**) Sucrose content. (**B**) Starch content. (**C**) SPS activity. (**D**) *StSPS* expression. (**E**) AGPase activity. (**F**) *StAGPase* expression. Different small letters represent significant differences (*p* < 0.05).

As shown in Figure 6A, in the transgenic plants, the starch content initially increased and then slightly decreased with the extension of the growth stage; in particular, the starch content of the *StBIN2*-overexpressing potatoes was significantly higher than that of the C10 potatoes at the 30th–60th day. The sucrose content decreased first and then increased (Figure 6B). After 40 days, the sucrose content in the overexpressing potatoes was significantly higher than in the wild-type potatoes. During the period of the concentrated growth and expansion of stolons, the overexpression of *StBIN2* significantly up-regulated sucrose and starch contents to prepare energy for tuber development.

As shown in Figure 6C, although the peak of SPS protein activity in the C10 potatoes appeared earlier than in the overexpression line, the enzyme activity in the *StBIN2* overexpression line was significantly higher than that of the wild type at the 40th–60th day. The peak of starch synthase activity was earlier in the transgenic line (Figure 6E) and was significantly higher than that of the C10 potatoes from the 20th to 40th day, suggesting significant differences in the early starch accumulation process. The expression of the sucrose phosphorylase gene (Figure 6D) was higher in the overexpressed material than in the wild type within 70 days, and the peak value appeared earlier than that of the wild type, while the *StAGPase* expression level (Figure 6F) was not significantly increased.

**Figure 7:**
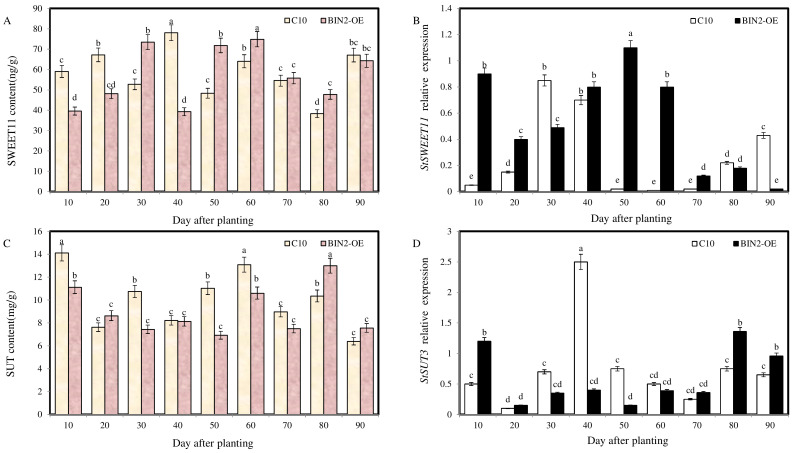
Effect of StBIN2 on sucrose transport during potato tuber formation. (**A**) SWEET11 protein content. (**B**) *StSWEET11* expression level. (**C**) SUT protein content. (**D**) *StSUT3* expression level. Different small letters represent significant differences (*p* < 0.05).

Figure 7 shows the expression levels and protein content of *SWEET11* and *SUT3*. The expression levels of the sucrose transporter increased when the top of the stolon was enlarged, and the expression levels of some sucrose transporter genes are always at a high level due to BIN2 overexpression, indicating that BIN2 can improve the expression levels of sucrose transporter genes, thereby promoting potato formation and providing energy for the process of potato formation. 

### 2.3. StBIN2 Physically Interacts in a Yeast Two-Hybrid System

**Figure 8:**
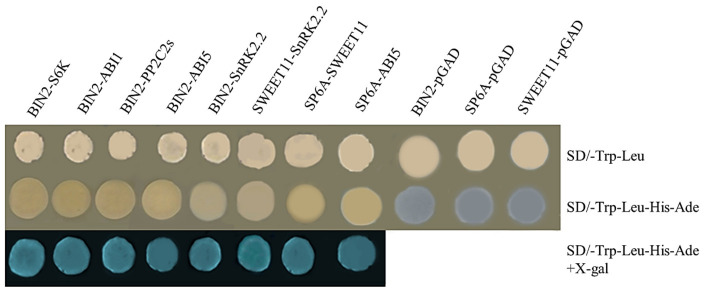
StBIN2 physically interacts in a yeast two-hybrid system. The proteins fused to pGADT7 are S6K, ABI1, PP2C2s, ABI5, SnRK2.2, and SWEET11, while the proteins fused to pGBKT7 are BIN2, SP6A, and SWEET11. X-gal—5-Bromo-4-chloro-3-indolyl-b-D-galactopyranoside acid. The experiments were repeated three times.

Previous studies have shown that BIN2 kinases are mainly involved in the regulation of plant growth and development through interactions with proteins [22,23,24,25,26,27,28,29,30,31,32]. BIN2 has been found to interact with many proteins of the ABA family in Arabidopsis [24]. Therefore, we performed a yeast two-hybrid assay and confirmed the interaction of BIN2-PP2C, BIN2-ABI5, BIN2-ABI1, BIN2-S6K2, BIN2-SnRK2.2, SP6A-SWEET11, SWEET11-SnRK2.2, SP6A-ABI1 (Figure 8). The interaction between proteins is likely to participate in BRs’ and ABA’s regulation of tuber formation through phosphorylation/dephosphorylation modifications. The above results also confirmed our conjecture that BIN2 does affect the ABA signaling pathway and potato-forming related proteins, thus affecting potato formation.

## 3. Discussion

BIN2 plays a central regulatory role in the BR signal pathway. In Arabidopsis, BR signaling plays a role in regulating chloroplast development by inhibiting the BIN2-mediated activation of GLKs [36]. BR-regulated root meristem development is mediated by the BIN2-UPB1 module [37]. BIN2 is also a hub connecting BRs and ABA. During seed germination, BIN2 phosphorylates and stabilizes ABI5 to mediate the ABA response, while BRs repress the BIN2-ABI5 cascade to antagonize ABA-mediated inhibition [23]. BIN2 also responds to drought, salt, and cold stresses by promoting key ABA signal transduction factors such as SnRK2s and PP2C [24,38]. BIN2 is the converging node that integrates sugar and BR signaling, and the sucrose-induced degradation of BIN2 leads to the accumulation of BZR1 and the enhancement of cellulose synthesis, thus promoting lateral root development [31]. In addition, BIN2 is also involved in disease resistance mediated by salicylic acid signaling, which induces the enhancement of BIN2 kinase activity and ultimately enhances the expression of disease-resistance genes [39]. However, the effect of BIN2 on yield has only been speculated to respond to stress resistance and thus maintain yield under stress. In the meantime, BIN2 has a regulating effect on plant growth and development (such as chlorophyll synthesis and root growth), which might also affect the yield.

The BR signaling pathway in *Arabidopsis thaliana* is clear. Firstly, BR signal transduction starts in BRI1, which regulates intracellular gene expression, phosphorylates BSK, and then affects BSU1 to inhibit BIN2. BIN2 binds to BZR1, which is phosphorylated by BIN2 and then transported into the cytoplasm via 14-3-3, and the overexpression of 14-3-3 can significantly alter SPS and SS activity [40,41]. In our study, during the critical period of tuber formation, BIN2 overexpression significantly enhanced SPS activity, which is consistent with the BIN2 regulatory pathway. Surprisingly, the expression of *SPS* also increased significantly, which may be regulated by other pathways. BIN2 does promote sucrose synthesis to provide material and energy support for tuber formation by promoting the activity of carbohydrate-synthesizing proteins and gene transcription.

**Figure A1:**
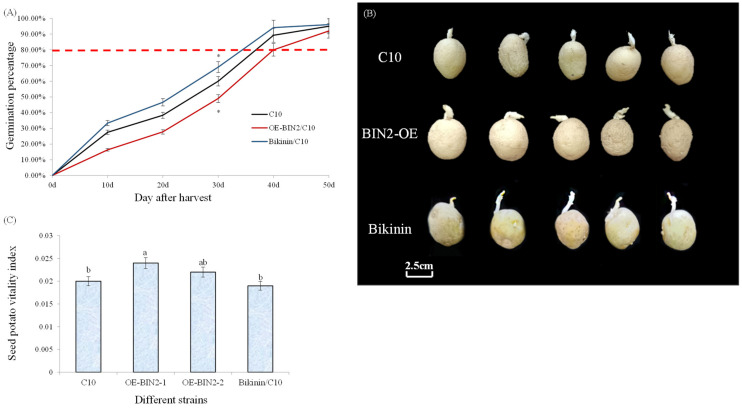
StBIN2 regulates potato bud elongation. (**A**) Germination rate of different potato lines. (**B**) Bud phenotype at 21 days after germination. (**C**) Seed potato viability index of different lines of potato. Different lowercase letters and * represent significant differences (*p* < 0.05). Bikinin, a reagent that promotes BR signaling, is an inhibitor of BIN2 [55].

In addition, we found that the overexpression of BIN2 resulted in inhibited germination and bud elongation and growth, but the buds were more robust, and the vitality of the seed potatoes was improved (Figure A1). In this experiment, the number of tubers produced by the per-basic seed was compared with that of the basic seed, but the data regarding the basic seed were not prominent for the following reasons: compared with the per-basic seed, the tuber itself in the control group had a large weight, so the multiple was not prominent. In addition, we did not conduct experiments with different sowing dates to match a seed potato viability index. In addition to the above, the exogenous application of certain concentrations of BRs can regulate the yield [42,43], which has an obvious concentration effect, and BIN2 may also have a concentration effect, that is, BIN2 overexpression lines with higher levels of expression may have higher or lower yield.

In addition to BRs and BIN2, BIN2 can also enhance ABA signaling by regulating ABA signal transduction factors such as ABI1, ABI5, and PP2C [23,24,25,26]. In this study, it was confirmed that in potato, BIN2 interacts with ABI1, ABI5, PP2C, and other proteins, and the function of BIN2 is most likely achieved through phosphorylation. Moreover, the expression levels of the genes *ABI1* and *ABI5* were up-regulated during the formation of potato tubers with the overexpression of *BIN2*, which is different from the result of promoting the yield reduction caused by an exogenous ABA application, indicating that the main role of this time was the downstream factor of ABA, namely the tuber-generating factor StSP6A. In the process of potato formation, BIN2 promoted the gene expression of *ABI1* and *ABI5*. In addition, the promotion of ABI5 can promote the protein activity of SP6A, a key factor in potato formation [24,34], which also explains that the promotion of the ABA signal increases the yield. In other words, BIN2’s regulation of hormone signals in the process of potato formation is not unitary but ultimately promotes the expression of key genes in potato tuber formation to promote potato formation. In addition, it has been reported that the interaction between SP6A and SWEET11 can promote its enzyme activity [35]. In this experiment, protein interaction in potatoes was confirmed, and the activity of SWEET protein in *StBIN2*-overexpressing potatoes was also significantly increased in the predominant tuber-expansion stage, which also promoted potato formation.

In summary, this study showed that the overexpression of *StBIN2* can, to a certain extent, promote potato formation and increase the number of tubers formed per plant and the weight of the tubers per plant. By promoting the enzyme activity and gene expression of the sugar transporter StSWEET11, StSUT, sucrose, and the starch-synthesizing proteins StSPS and StAGPase, StBIN2 affects the conversion and utilization of key substances such as glucose, sucrose, and starch, thus providing a material basis and energy preparation for potato formation. In addition, the interaction between StBIN2 and the ABA signaling factor StABI5 promotes the downstream transmission of ABA signals, thus promoting the expression of the tuber-formation factor StSP6A, and SP6A can promote the sugar transporter StSWEET11, jointly promoting potato formation. On the basis of previous research, this experiment verified the phenotype and explored the mechanism of BIN2 overexpression in potato, providing a theoretical basis for molecular breeding to guide production. It is worth noting that this study mainly focused on the tuber formation process and analyzed the phenotype, physiological and biochemical indexes, and gene expression levels of seed potato but did not pay special attention to plant growth and stress resistance, which might be explored in a future investigation.

## 4. Materials and Methods

### 4.1. Gene and Protein Structure Analysis

A protein subcellular localization prediction was performed using WoLF PSORT and YLoc (http://www.genscript.com/wolf-psort.html, accessed on 6 May 2022) [44]. From the NCBI (https://www.ncbi.nlm.nih.gov/, accessed on 7 May 2022), the BIN2 protein sequences of 15 other plants, including pepper, Arabidopsis, and tomato, were downloaded and compared with amino acid sequences, using DNAMAN 7.212 (Lynnon Biosoft, QC, Canada). The StBIN2 protein’s tertiary structure was predicted using SWISS-MODEL (https://swissmodel.expasy.org/, assessed on 1 November 2023). The phylogenetic tree was constructed using the proximity method in MEGA 11 software (Temple University, Philly, PA, USA) [45]. The gene structure was analyzed online via GSDS. In PGSC (https://spuddb.uga.edu/, accessed on 9 March 2021), the 1500 bp upstream sequence of the *StBIN2* initiation codon ATG was obtained as the promoter sequence. PlantCARE (http://bioinformatics.psb.ugent.be/webtools/plantcare/html/, accessed on 7 May 2022) was used to analyze the cy-acting elements of the promoter region [46].

### 4.2. Plant Material and Growth Conditions

The experimental material was the tetraploid potato variety “Chuanyu 10” (C10), commonly used in Sichuan Province, which is a medium and early maturing variety bred by the Crop Research Institute of the Sichuan Academy of Agricultural Sciences. The virus-free tissue culture seedlings of this variety were provided by the Potato Research and Development Center of Sichuan Agricultural University.

Tissue culture technology was used to expand potato tissue culture seedlings. The seedlings were cultured in 60 mm glass bottles containing 15 g/L of sucrose and 7 g/L of agar and placed in a tissue culture room at 24 °C with a 16 h/8 h light and dark cycle. The tissue culture seedlings were cultured for 25 days, washed with agar, cut to remove excess roots, and transplanted into the matrix greenhouse.

### 4.3. The Generation of StBIN2 Transgenic Potato Lines

To generate *StBIN2* constructs for overexpressing transgenic potato lines, primers were designed to amplify a 1149 bp ORF (open read frame) from tuber cDNA prepared from the potato cultivar “Chuanyu 10”. *Xba*I and *Sma*I sites were added at the starts and the termini, respectively, and the primers were designed as 5′-GATCggatccATGGCTGATGATAAGGAGATGTC-3′ and 5′-GATCcccgggCACGTCATGTCATCACGGG-3′. The recombinant vector pBI121-BIN2 was constructed with T4 ligase and then digested using restriction enzymes, as described above, for a determination of the accuracy. Similarly, a 1149 bp StBIN2-specific fragment was amplified via PCR and used in a restriction-ligation reaction for insertion into the binary vector pBI121. The recombinant expression vectors were transformed into *Rhizobium radiobacter* (*Agrobacterium tumefaciens*) GV3101 via the freeze–thaw method. The stem segments of the 7-day in vitro culture were infected and transformed as described previously [47]. Finally, aseptic plantlets with different expression levels of *StBIN2* were obtained.

### 4.4. Sampling and Processing Methods

Tissue expression analysis: The roots, stems, top new leaves, flowers, and stolons of potato tissue culture seedlings were transplanted for 20 d and the eyes and pith of a potato tuber harvested for 10 d were sampled for a tissue expression analysis. 

Plant and stolon determination: Ten days after the tissue culture seedlings were planted, the tops of the potato plants with diameters of 4 mm and 3 mm were randomly taken every ten days. Tissues less than 3–4 mm were taken according to the original sample of the stolon, and 10 plants were sampled each time until the end of the growth period. The number and weight were measured.

Seed potato harvest: At the end of the growth period, the number of tubers per plant and the weight of the tubers were measured. The number of tubers per plant and the weight of the tubers were calculated according to the following formula: the number of tubers per plant (per plant) = the total number of tuber plants sampled/the number of plants sampled. Tuber weight per plant (g/plant) = total tuber weight of the sampled plants/number of sampled plants.

### 4.5. RNA Extraction and cDNA synthesis

Total RNA was extracted using a SteadyPure universal RNA extraction kit (Ecorry, Changsha, China), RNA integrity was determined via 1% agarose gel electrophoresis, and the concentration of RNA was determined using a Nano Drop One (Thermo Scientific, Waltham, MA, USA). Using 1µg of total RNA as template, an Evo M-ML V reverse transcription kit (Ecorui, Changsha, China) was used for reverse transcription and cDNA synthesis [48].

### 4.6. Index Measurement

For the sucrose content determination method, refer to Zarbakhsh’s method [49]. The starch content was determined via the anthrone colorimetric method. The activities of SWEET11, SUT3, SPS, AGPase, BIN2, and other enzymes were all detected via an enzyme-linked immunosorbent assay [50,51].

### 4.7. Quantitative Real-Time PCR

The tissue expression and induced expression characteristics of *BIN2* were determined via a real-time PCR (Roche, Rotkreuz, Switzerland) for the identification of BIN2 transgenic lines. The reaction system (20 µL) consisted of 10 µL of Trans Start Top Green qPCR Super Mix (Full gold, Beijing, China), a 1.2 µL cDNA template, 0.5 µL (10 µmol/L) of each the PCR upstream and downstream primers, and 7.8 µL of ddH_2_O. The qRT-PCR reaction procedure specifications were as follows: 95 °C 5 s, 56 °C 10 s, 72 °C 10 s, 50 cycles. Using *EF1ɑL* as the internal reference gene, the relative gene expression was calculated via the 2^−ΔΔCt^ method [52].

### 4.8. Yeast Two-Hybrid Assay

The full length of *BIN2* was fused to the bait vector pGBKT7, and the AD vector (pGADT7) was used to express others. Then, both pairs of plasmids (such as BIN2-BD/ABI5-AD) were co-transformed into the yeast strain AH109 (Coolaber, Hangzhou, China). The co-transformation colonies were selected on SD-Trp-Leu plates. Positive clones were transferred and grown on SD/-Leu-Trp-His plates, and the β-Galactosidase activity was measured. BIN2-BD and pGADT7 were used as negative controls. All proteins were used as positive controls [53].

### 4.9. Statistical Analysis

For all generated data, at least three biological replicates were performed for each sample. The data were subjected to unpaired Student’s *t*-tests, with *p* ≤ 0.01 and *p* ≤ 0.05. Data are shown as means ± SEs (*n* = 3), and n represents the biological replicates. Excel 2010 (Microsoft Corporation, Redmond, WA, USA) and SPSS 14.0 software (IBM, New York, NY, USA) were used for the statistical analysis. The statistical results were reported as means ± SDs [54].

## Figures and Tables

**Table 1:** Some important *cis*-acting regulatory elements in the promoters of *StBIN2.*

*Cis*-Element	Core Sequence	Functional Description of *Cis*-Element	Positions
ABRE	ACGTG	*cis*-acting element involved in abscisic acid responsiveness	−50
TGA-box A	TGACGTA	part of an auxin-responsive element	−23
TGA-element	AACGAC	auxin-responsive element	−1296
TATC-box	TATCCCA	*cis*-acting element involved in gibberellin responsiveness	−643, −871
P-box	CCTTTTG	gibberellin-responsive element	−64, −82, −512
CGTCA-motif	CGTCA	*cis*-acting regulatory element involved in the MeJA responsiveness	−23
TCA-element	CCATCTTTTT	*cis*-acting element involved in salicylic acid responsiveness	−824
CAT-box	GCCACT	*cis*-acting regulatory element related to meristem expression	−250, −263, −1521
WUN-motif	AAATTTCCT	wound-responsive element	−726
ARE	AAACCA	*cis*-acting regulatory element essential for the anaerobic induction	−1283
MYB	TAACCA	MYB transcription factor binding site	−28, −533, −1220, −1330
MYC	CATGTG	MYC transcription factor binding site	−56, −1170
MYC	CATTTG	MYC transcription factor binding site	−867, −1320
GT1-motif	GGTTAA	light responsive element	−534, −1329
G-box	CACGTC	*cis*-acting regulatory element involved in light responsiveness	−49
Box 4	ATTAAT	part of a conserved DNA module involved in light responsiveness	−892, −911, −997, −1126

## Data Availability

The data presented in this study are available within the article.
